# Higher clinical success in patients with ventilator-associated pneumonia due to methicillin-resistant *Staphylococcus aureus* treated with linezolid compared with vancomycin: results from the IMPACT-HAP study

**DOI:** 10.1186/cc13914

**Published:** 2014-06-10

**Authors:** Paula Peyrani, Timothy L Wiemken, Robert Kelley, Marcus J Zervos, Daniel H Kett, Thomas M File Jr, Gary E Stein, Kimbal D Ford, Ernesto G Scerpella, Verna Welch, Julio A Ramirez

**Affiliations:** 1 Division of Infectious Diseases, University of Louisville and VA Medical Center, 501 E. Broadway, Louisville, KY 40202, USA; 2 Henry Ford Health System, One Ford Place, Detroit, MI 48202, USA; 3 University of Miami Miller School of Medicine, 1601 NW 12th Avenue, Miami, FL 33136, USA; 4 Jackson Memorial Hospital, 1601 NW 12th Avenue, Miami, FL 33136, USA; 5 Summa Health System, 29 North Adams Street, Akron, OH 44304, USA; 6 Michigan State University, 220 Trowbridge Road, East Lansing, MI 48824, USA; 7 Infectious Diseases, Specialty Care Medicines Development Group, Pfizer Inc., 500 Arcola Road, Collegeville, PA 19426, USA

## Abstract

**Introduction:**

Controversy exists regarding optimal treatment for ventilator-associated pneumonia (VAP) due to methicillin-resistant *Staphylococcus aureus* (MRSA). The primary objective of this study was to compare clinical success of linezolid versus vancomycin for the treatment of patients with MRSA VAP.

**Methods:**

This was a multicenter, retrospective, observational study of patients with VAP (defined according to Centers for Disease Control and Prevention criteria) due to MRSA who were treated with linezolid or vancomycin. MRSA VAP was considered when MRSA was isolated from a tracheal aspirate or bronchoalveolar lavage. Clinical success was evaluated by assessing improvement or resolution of signs and symptoms of VAP by day 14. After matching on confounding factors, logistic regression models were used to determine if an association existed between treatment arm and clinical success.

**Results:**

A total of 188 patients were evaluated (101 treated with linezolid and 87 with vancomycin). The mean ± standard deviation Acute Physiology and Chronic Health Evaluation (APACHE) II score was 21 ± 11 for linezolid- and 19 ± 9 for vancomycin-treated patients (*P* = 0.041). Clinical success occurred in 85% of linezolid-treated patients compared with 69% of vancomycin-treated patients (*P* = 0.009). After adjusting for confounding factors, linezolid-treated patients were 24% more likely to experience clinical success than vancomycin-treated patients (*P* = 0.018).

**Conclusions:**

This study adds to the evidence indicating that patients with MRSA VAP who are treated with linezolid are more likely to respond favorably compared with patients treated with vancomycin.

## Introduction

Methicillin-resistant *Staphylococcus aureus* (MRSA) is one of the primary multidrug-resistant pathogens causing healthcare-associated infections in the United States [1]. Healthcare-associated respiratory infections caused by MRSA, including healthcare-associated pneumonia (HCAP), hospital-acquired pneumonia (HAP), and ventilator-associated pneumonia (VAP), are important causes of morbidity and mortality [2]. National guidelines for the management of these patients were published by the American Thoracic Society (ATS)/Infectious Diseases Society of America (IDSA) in 2005 [2]. The guidelines placed vancomycin and linezolid at similar levels of efficacy and recommended either antibiotic as appropriate therapy.

The results of the only prospective, randomized, double-blind clinical trial exclusively enrolling patients with MRSA healthcare-associated respiratory infections that compared the clinical efficacy of vancomycin versus linezolid were published in 2012, well after the release of the national guidelines [3]. In this trial, linezolid-treated patients had a higher clinical success rate compared with vancomycin-treated patients. Although the sub-analysis of patients with VAP in this study indicated a 55% clinical success rate for linezolid and a 46% clinical success rate for vancomycin, this difference was not statistically significant [3]. In the field of critical care medicine, this study did not completely resolve the controversy regarding optimal therapy for patients with MRSA VAP.

The Improving Medicine through Pathway Assessment of Critical Therapy in Hospital-Acquired Pneumonia (IMPACT-HAP) study was created in 2006 by a group of clinical investigators interested in healthcare-associated respiratory infections [4,5]. Considering the persistent controversy regarding the treatment of MRSA VAP, the IMPACT-HAP group performed the current study with the primary objective of comparing clinical success rates for patients with MRSA VAP who were treated with linezolid or vancomycin. As secondary study objectives, mortality, safety, and resource utilization were compared between the two groups.

## Methods

### Study design and study sites

IMPACT-HAP was a multicenter, retrospective, observational study of intensive care unit (ICU) patients with MRSA VAP who were treated with linezolid or vancomycin. The following five tertiary academic medical centers in the United States participated in the study: the University of Louisville Medical Center (Louisville, KY, USA), Henry Ford Health System (Detroit, MI, USA), University of Miami/Jackson Memorial Hospital (Miami, FL, USA), Summa Health System (Akron, OH, USA), and Michigan State University (East Lansing, MI, USA). The study was conducted from November 2008 through October 2012. Patient data were collected on a case report form, entered into a web-based database, and transferred electronically to the University of Louisville Clinical and Translational Research Support Center for data validation and quality. The study was approved by the institutional review board at each participating institution (University of Louisville Human Subjects Protection Program Office; Summa Health System Institutional Review Board; Michigan State University Human Research Protection Program; Henry Ford Health System Institutional Review Board; University of Miami Human Subjects Research Office), all of which waived the requirement for informed consent since this was a retrospective observational study.

### Study population and measurements

#### Inclusion criteria

Adult patients in participating ICUs meeting the study definition of MRSA VAP were eligible for inclusion. VAP was defined according to the Centers for Disease Control and Prevention (CDC) National Healthcare Safety Network surveillance definitions [6]. VAP was considered to be due to MRSA when isolated from tracheal aspirates, bronchoalveolar lavage obtained by bronchoscopy, or blinded bronchoalveolar lavage. Patients must have received more than 48 hours of either vancomycin or linezolid.

#### Exclusion criteria

Patients with a ‘comfort care’ or ‘do not resuscitate’ order and patients who developed clinical failure during the initial 48 hours of antibiotic therapy were excluded from the study. In clinical practice, ICU physicians change antibiotics due to their own preferences without the evidence of patients’ clinical deterioration. Patients were also excluded if there was a switch from vancomycin to linezolid or vice versa after 48 hours in a patient without evidence of clinical failure.

#### Study variables

At the time of clinical diagnosis of VAP (day 0), data on patients’ demographic and baseline characteristics, severity of illness including Acute Physiology and Chronic Health Evaluation (APACHE) II score and Clinical Pulmonary Infection Score, diagnostic procedures, and treatment were collected. While hospitalized, patients were followed until discharge, death, or 28 days after VAP diagnosis, whichever occurred first. Laboratory values were collected during hospitalization. Identification of MRSA isolates and *in vitro* susceptibility were performed at each participating center. Vancomycin serum trough levels were collected throughout the study period.

### Study outcomes

#### Primary study outcome

*Clinical Success:* defined as improvement or resolution of the signs and symptoms of VAP. This outcome was evaluated at day 14 or hospital discharge, whichever occurred first. To be considered a clinical success, patients must have received at least five days of either vancomycin or linezolid.

#### Secondary study outcomes

*Mortality*: defined as all-cause mortality within 14 days after VAP diagnosis.

*Thrombocytopenia:* defined as a platelet count <150,000 cells/mm^3^ or a 50% decrease in platelet count if low at baseline. Platelet count within 24 hours of VAP diagnosis was defined as the baseline value.

*Anemia:* defined as hemoglobin ≤10 g/dL or a 2 g/dL decrease if low at baseline. Hemoglobin level within 24 hours of VAP diagnosis was defined as the baseline value.

*Nephrotoxicity:* defined as an increase in serum creatinine of 0.5 mg/dL or 50% above baseline, whichever was greater, in ≥2 consecutive measurements. Serum creatinine level within 24 hours of VAP diagnosis was defined as the baseline value.

#### Resource utilization

For each study group, resource utilization was evaluated by the following outcomes: 1) days on mechanical ventilation, calculated as the number of days from VAP diagnosis to extubation or to discharge if not extubated; 2) length of stay (LOS) in the ICU, calculated as the number of days from VAP diagnosis to discharge from the ICU; and 3) LOS in the hospital, calculated as the number of days from VAP diagnosis to discharge from the hospital.

### Statistical analysis

Categorical variables were expressed as frequencies and percentages and were compared between the treatment groups using chi-squared or Fisher’s exact tests. Continuous variables were expressed as medians and interquartile ranges or means and standard deviations and were compared between groups using the Mann-Whitney *U* test or the Student’s *t* test. *P* values ≤0.05 were considered statistically significant in all analyses unless otherwise specified.

#### Clinical success

In-depth methods for comparing clinical success between the treatment groups are explained in Additional file 1. Briefly, we used absolute standardized differences and relative effect statistics to assess for covariate imbalances between patients treated with vancomycin and linezolid [7]. This allowed us to evaluate small imbalances in the data that other traditional methods of confounding identification may have missed. To correct for imbalances (for example, confounding effects), we used a multivariate matching algorithm to match each patient treated with linezolid to one patient treated with vancomycin who was most similar with respect to all confounding variables analyzed [8]. This produced a matched dataset for which we re-assessed the covariate imbalance again using the absolute standardized difference and relative effect statistics. Any variables that remained imbalanced between the treatment groups were included as covariates in a final logistic regression model.

We also performed sensitivity analyses using other methods of covariate balance. A description of these methods also can be found in Additional file 1. Since the odds ratio (OR) generated from this logistic regression model may be a poor descriptor of the actual risk when the prevalence of the outcome is high (for example, high rates of clinical success), the OR was adjusted to reflect the relative risk of obtaining the outcome [9]. Using results from these models, the adjusted number needed to treat was calculated according to published procedures [10].

A propensity score containing all variables that were unbalanced according to the unmatched absolute standardized difference calculation described previously was created. The propensity score was included in a final logistic regression model to adjust for confounding effects between the treatment and outcome. The predicted probabilities of clinical success at day 14 were plotted in a line graph against the APACHE II score for each treatment group.

#### Safety

Kaplan-Meier survival curves were used to examine the statistical differences between linezolid and vancomycin treatment in the time to each of the safety outcomes (thrombocytopenia, anemia, nephrotoxicity). The log-rank test was used to assess statistical significance.

#### Resource utilization

Kaplan-Meier survival curves were created to evaluate the bivariate relationships between treatment groups and resource utilization (time on mechanical ventilation, LOS in the ICU, and LOS in the hospital).

#### Statistical software

R software version 2.15.1 was used for all analyses including the following packages: MatchIt [8], matching [11], nonrandom [12], rgenoud [13], car [14], Zelig [15], Cairo [16], survival [17], and Hmisc [18].

## Results

### Patient characteristics

A total of 188 patients diagnosed with MRSA VAP were included in this analysis, of whom 101 were treated with linezolid and 87 with vancomycin. A comparison of baseline characteristics between the treatment groups is depicted in Table 1. Linezolid-treated patients were significantly more likely to be hospitalized for ≥5 days before VAP diagnosis and to have bronchiectasis, end-stage liver disease, or severe sepsis. Linezolid-treated patients also had significantly higher APACHE II scores and significantly lower hemoglobin levels at diagnosis. The rate of *Clostridium difficile* infection in each arm was as follows: linezolid arm, 12 (12%); vancomycin arm 11 (13%); *P* = 0.873. Additional results associated with methods described in Additional file 1 can be found in Additional files 2, 3, and 4.

**Table 1:** **Baseline characteristics of adult intensive care unit patients with ventilator-associated pneumonia due to methicillin-resistant *Staphylococcus aureus *** **treated with linezolid or vancomycin**

**Characteristic**	**Linezolid (n = 101)**	**Vancomycin (n = 87)**	***P *** **value**
Male, n (%)	63 (62.4)	49 (56.3)	0.457
Hospitalization ≥5 days before therapy for VAP, n (%)	74 (73.3)	50 (57.5)	0.030
Bronchiectasis, n (%)	8 (7.9)	1 (1.1)	0.039
Colonization with MDRO, n (%)	34 (33.7)	32 (36.8)	0.759
Hospitalization ≥2 days in previous 90 days, n (%)	20 (19.8)	17 (19.5)	1.000
Nursing home resident, n (%)	6 (5.9)	5 (5.7)	1.000
Home infusion therapy, n (%)	2 (2.0)	0	0.500
Home wound care, n (%)	5 (5.0)	3 (3.4)	0.727
Active malignancy, n (%)	8 (7.9)	5 (5.7)	0.774
End-stage liver disease, n (%)	9 (8.9)	1 (1.1)	0.022
COPD, n (%)	15 (14.9)	8 (9.2)	0.271
Steroid use, n (%)	4 (4.0)	7 (8.0)	0.351
Risk factors for MDROs, n (%)	88 (87.1)	70 (80.5)	0.236
Cardiac disease, n (%)	29 (28.7)	25 (28.7)	1.000
Renal disease, n (%)	8 (7.9)	6 (6.9)	1.000
Vascular disease, n (%)	20 (19.8)	27 (31.0)	0.092
End-stage renal disease and/or dialysis, n (%)	4 (4.0)	3 (3.4)	1.000
Diabetes, n (%)	29 (28.7)	17 (19.5)	0.174
Respiratory disease, n (%)	24 (23.8)	18 (20.7)	0.726
Multilobar infiltrates, n (%)	39 (38.6)	25 (28.7)	0.168
Severe sepsis, n (%)	78 (77.2)	55 (63.2)	0.038
Appropriate empiric antimicrobial therapy, n (%)	100 (99.0)	85 (97.7)	0.597
Age, median (IQR)	59 (20)	56 (26)	0.246
Body mass index, median (IQR)	28.7 (11.9)	27.7 (9.9)	0.359
CPIS at diagnosis, median (IQR)	6 (3)	6 (2)	0.177
CPIS at day 3, median (IQR)	7 (3)	7 (3)	0.051
APACHE II score, median (IQR)	21 (11)	19 (9)	0.041
Platelet count at diagnosis, median (IQR)	219 (143)	204 (115.5)	0.397
Hemoglobin at diagnosis, median (IQR)	9.5 (1.9)	10 (2.1)	0.026
Creatinine clearance at diagnosis, median (IQR)	78.5 (59.3)	95.9 (69.3)	0.054
Vancomycin MIC (μg/mL, E-test), n (%)			0.087
0.75	1 (2.4)	0	
1	5 (11.9)	0	
1.5	22 (52.4)	26 (72.2)	
2	14 (33.3)	10 (27.8)	
Vancomycin serum trough level (μg/mL),			
mean ± SD			
Day 3	…	13 ± 8	
Overall	…	21 ± 11	

APACHE, Acute Physiology and Chronic Health Evaluation; COPD, chronic obstructive pulmonary disease; CPIS, Clinical Pulmonary Infection Score; IQR, interquartile range; MDRO, multidrug-resistant organism; MIC, minimum inhibitory concentration; VAP, ventilator-associated pneumonia.

MRSA minimum inhibitory concentrations (MICs) to vancomycin also are depicted in Table 1. The mean ± standard deviation (SD) vancomycin trough level at day 3 was 13 ± 8 μg/mL for vancomycin-treated patients. The mean ± SD duration of treatment was 11 ± 4 days for linezolid- and 11 ± 5 days for vancomycin-treated patients (*P* = 0.512).

### Primary study outcome: clinical success

#### Unadjusted

The unadjusted clinical success rates for each study arm were as follows: linezolid, 86/101 (85%); vancomycin, 60/87 (69%; *P* = 0.009).

#### Propensity adjusted

**Figure 1:**
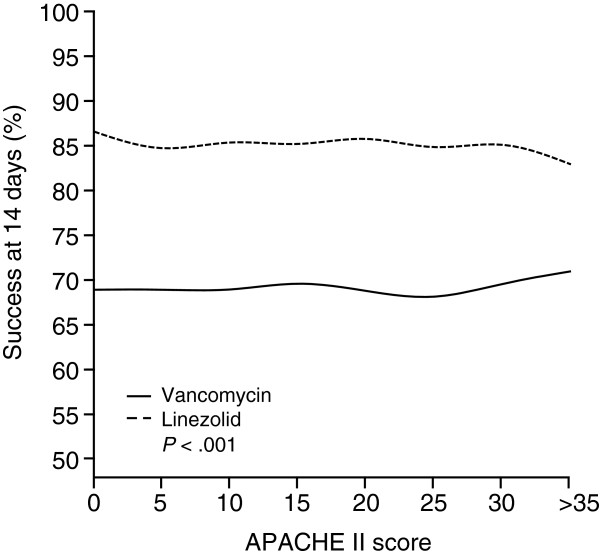
Propensity-adjusted logistic regression model for clinical success at day 14 across a range of Acute Physiology and Chronic Health Evaluation (APACHE) II scores.

Figure 1 shows the results of the propensity-adjusted regression model for clinical success at day 14. After adjusting for confounding effects, linezolid-treated patients had statistically significantly higher clinical success rates than vancomycin-treated patients. These results were consistent across a wide range of APACHE II scores.

#### Multivariate matching

The results of the final logistic regression model, after matching, adjustment for the remaining covariate imbalances, and adjustment of the OR to reflect relative risk, indicated that linezolid-treated patients were 24% more likely to reach clinical success by day 14 than vancomycin-treated patients (relative risk, 1.24; 95% confidence interval (CI), 1.06 to 1.32; *P* = 0.018; adjusted from: OR, 3.53; 95% CI, 1.25 to 9.99; *P* = 0.018). The adjusted number needed to treat with linezolid to achieve one extra case of clinical success compared with vancomycin was six.

### Secondary study outcomes

A comparison of unadjusted secondary study outcomes in the two treatment groups is depicted in Table 2.

**Table 2:** Unadjusted secondary outcomes

**Outcome**	**Linezolid (n = 101)**	**Vancomycin (n = 87)**	***P *** **value**
Mortality, n (%)	10 (9.9)	8 (9.2)	1.00
Thrombocytopenia, n (%)	18 (17.8)	16 (18.4)	1.00
Anemia, n (%)	43 (42.6)	41 (47.1)	0.559
Nephrotoxicity, n (%)	11 (10.9)	13 (14.9)	0.541
Days on mechanical ventilation, median (IQR)	11 (14)	13 (11.5)	0.276
Length of stay in the ICU, median (IQR)	11 (14)	13 (11.5)	0.823
Length of stay in the hospital, median (IQR)	18 (19)	16 (14.5)	0.773

ICU, intensive care unit; IQR, interquartile range.

#### Mortality

A total of 18/188 (10%) of patients died. No statistically significant differences were found between the treatment groups with respect to mortality.

#### Safety

**Figure 2:**
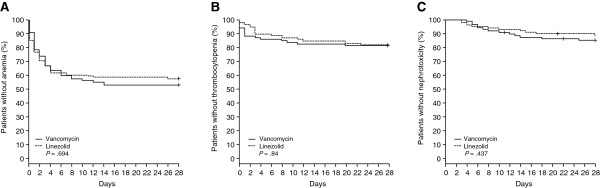
**Kaplan-Meier survival curves for each study arm. (A)** Anemia. **(B)** Thrombocytopenia. **(C)** Nephrotoxicity.

Figure 2 depicts Kaplan-Meier survival curves for anemia, thrombocytopenia, and nephrotoxicity using the unmatched dataset. No statistically significant differences were found between the treatment groups with respect to any of the three safety outcomes.

#### Resource utilization

**Figure 3:**
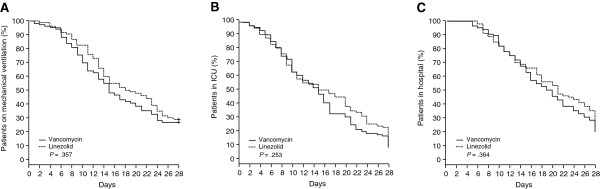
**Kaplan-Meier survival curves for each arm. (A)** Days on mechanical ventilation. **(B)** Length of stay (LOS) in the intensive care unit (ICU). **(C)** LOS of stay in the hospital.

Figure 3 depicts Kaplan-Meier survival curves for days on mechanical ventilation, LOS in the ICU, and LOS in the hospital using the unmatched dataset. No statistically significant differences were found between the treatment groups with respect to any of the three resource utilization outcomes.

## Discussion

The results of this study indicate that patients with MRSA VAP who are treated with linezolid have a significantly higher clinical success rate compared with patients treated with vancomycin. This study did not identify any significant differences between linezolid- and vancomycin-treated patients with respect to mortality, development of thrombocytopenia, anemia, or nephrotoxicity, mechanical ventilator days, or length of ICU or hospital stay.

Patients with MRSA VAP who were treated with linezolid were approximately 15% more likely to reach clinical success when compared with vancomycin-treated patients. Six patients with MRSA VAP need to be treated with linezolid to achieve one additional clinical success. The difference in clinical success rates between treatment groups in our study is consistent with the 11% higher clinical success rate for linezolid-treated patients recently reported in a prospective, double-blind trial [3].

The clinical success rate of 85% observed among linezolid-treated patients in our study is high compared with success rates reported in previous studies [19-23]. This may be due to our exclusion of patients with a ‘comfort care’ or ‘do not resuscitate’ order and patients who developed clinical failure during the initial 48 hours of antibiotic therapy.

There are several reasons why patients treated with linezolid may have been more likely to reach clinical success in our study. First, linezolid-treated patients might have had less severe disease. Second, vancomycin-treated patients might have had suboptimal vancomycin trough levels. Third, the MRSA MIC to vancomycin might have been in the upper limit within the susceptible range. Finally, linezolid may be a more effective antibiotic than vancomycin for the treatment of MRSA VAP.

The possibility that linezolid-treated patients had less severe disease is an unlikely explanation for our findings. Linezolid-treated patients had a significantly higher mean APACHE II score and a significantly higher proportion had developed severe sepsis at the time of VAP diagnosis. This imbalance in the severity of disease between patients treated with linezolid and vancomycin was mitigated through the use of a multivariable matching algorithm.

The possibility of suboptimal levels in vancomycin-treated patients in our study is a consideration. Vancomycin trough levels of 15 to 20 μg/mL may result in better therapeutic outcomes in patients with VAP than conventional trough levels of 5 to15 μg/mL [2]. In our study, clinical pharmacists were actively involved in the dosing and management of vancomycin as part of each institution’s antimicrobial stewardship program. Vancomycin-treated patients had a mean trough level of 13 ± 8 μg/mL at day 3. We also collected the highest or maximum trough level at any point during the treatment period. The mean ± SD of the maximum vancomycin trough level throughout the treatment duration was 21 ± 11 μg/mL.

An earlier publication from the IMPACT-HAP study group reported that mortality among patients with MRSA HCAP, HAP, and VAP increased as a function of vancomycin MIC [24]. Of the 78 patients in the current study for whom vancomycin MICs were available, the majority (72 patients) had vancomycin MICs >1 μg/mL. This may explain the lower success rates seen in the vancomycin group.

Finally, linezolid may be a more effective antibiotic than vancomycin for the treatment of MRSA VAP. It also has been hypothesized that the better lung penetration of linezolid may in part explain the improved outcomes seen in patients with MRSA VAP [25]. However, our study cannot address this hypothesis.

The mortality rate in our population was exceedingly low (10%). However, we identified several reasons that may explain a low mortality rate in patients with VAP. First, patients with a ‘comfort care’ or ‘do not resuscitate’ order were excluded from the study. Second, patients who developed clinical failure during the initial 48 hours of antibiotic therapy were also excluded. Third, we only evaluated mortality up to day 14. These three factors excluded patients with high disease severity and early mortality as well as patients with late mortality. The low mortality rate found in this study limits the power to detect any statistical or clinical differences in mortality between treatment groups. Moreover, because it has been suggested that the attributable mortality of VAP is <10% [26-28], the statistical power to detect differences in VAP-related mortality is even lower. This low attributable mortality and the patient’s primary diagnosis requiring mechanical ventilation make it difficult to ascertain differences in outcomes beyond clinical success in patients with VAP. It should be kept in mind that the low mortality observed in our study might be partially due to patients meeting the CDC criteria for VAP, but in reality having an alternative diagnosis.

The findings of this study indicate that anemia, thrombocytopenia, and nephrotoxicity are frequent events in ICU patients with MRSA VAP. However, there were no statistically significant differences in the incidence of these outcomes in ICU patients with MRSA VAP who were treated with linezolid or vancomycin. The hematologic and renal deterioration seen in patients with MRSA VAP appear independent of antibiotic choice and may be caused by other conditions common in the critically ill.

We found no difference in resource utilization between patients with MRSA VAP who were treated with linezolid versus vancomycin. The large variability of resource utilization among ICU patients [29] may explain why our study failed to detect a difference between treatment groups. To develop interventions to control resource utilization and the cost of care among patients with VAP, factors beyond appropriate antibiotic therapy should be explored.

Our study has several significant limitations. First, this study was observational in design, which has inherent limitations when compared to randomized clinical trials. Second, the VAP diagnosis was based on CDC surveillance criteria, which may misclassify some cases. The optimal clinical diagnosis of VAP based on quantitative cultures obtained from bronchoalveolar lavage was performed in a limited number of patients. Since most of the microbiology was obtained from endotracheal aspirates, we should emphasize that misclassification of microbiological etiology may have occurred in a significant number of patients. Third, the follow-up period of the study was up to 14 days after VAP diagnosis, which limited our ability to evaluate outcomes occurring at a later time. The lack of evaluation of mortality at 30 days weakens our conclusions. Fourth, although clinical pharmacists were involved in the management of vancomycin pharmacokinetics, the optimal vancomycin trough level at day 3 was not achieved in a number of patients. This observational study represents patients from ICUs in tertiary care, university-affiliated institutions, which may limit the generalizability of our study. Finally, we recognize that we failed to collect information on some relevant clinical features such as: acute and chronic tracheostomy, condition and disposition at hospital discharge, readmission rates, and eradication of MRSA colonization*.*

## Conclusions

In conclusion, this study adds to the evidence indicating that patients with MRSA VAP who are treated with linezolid are more likely to respond favorably compared with patients treated with vancomycin. These results suggest that linezolid should be considered a preferred antibiotic for treatment of patients with MRSA VAP.

## Key messages

• Patients with MRSA VAP who are treated with linezolid have a significantly higher clinical success rate compared with patients treated with vancomycin.

• Patients with MRSA VAP who were treated with linezolid were approximately 15% more likely to reach clinical success when compared with vancomycin-treated patients.

• This study did not identify any significant differences between linezolid- and vancomycin-treated patients with respect to development of thrombocytopenia, anemia, or nephrotoxicity.

• Resource utilization, measured by days on mechanical ventilator, or length of ICU or hospital stay was not different for patients treated with linezolid or vancomycin.

## Abbreviations

APACHE: Acute Physiology and Chronic Health Evaluation; ATS: American Thoracic Society; CDC: Centers for Disease Control and Prevention; CI: confidence interval; COPD: chronic obstructive pulmonary disease; CPIS: Clinical Pulmonary Infection Score; HAP: hospital-acquired pneumonia; HCAP: healthcare-associated pneumonia; ICU: intensive care unit; IDSA: Infectious Diseases Society of America; IMPACT-HAP: Improving Medicine through Pathway Assessment of Critical Therapy in Hospital-Acquired Pneumonia; IQR: interquartile range; LOS: length of stay; MDRO: multidrug-resistant organism; MICs: minimum inhibitory concentrations; MRSA: methicillin-resistant *Staphylococcus aureus*; OR: odds ratio; SD: standard deviation; VAP: ventilator-associated pneumonia.

## Competing interests

PP received travel funds and grant support from Pfizer Inc. TLW and RK received grant support from Pfizer Inc. MJZ received grants from Pfizer Inc., Cubist, and Cerexa; received a speaker honorarium from Sunovion; and was a consultant for Optimer. DHK served as a consultant for Pfizer Inc. and Astellas; received honoraria or speaking fees from Pfizer Inc., Astellas, and GlaxoSmithKline; and received grant support from Pfizer Inc. TMF received grants from Summa Health System, Pfizer Inc., and Forest; served as a consultant for Bayer, Cubist, Daiichi Sankyo, and GlaxoSmithKline; and served on advisory boards for Durata, Merck, and Tetraphase. GES received grant support from and served as a consultant for Pfizer Inc. KDF and VW are employees and shareholders of Pfizer Inc. EGS, formerly of Pfizer Inc., was an employee and shareholder of Pfizer Inc. at the time this manuscript was developed. JAR is on the speaker bureau, is a consultant, and received research support from Pfizer Inc.

## Authors’ contributions

All authors have made substantial contributions to conception and design, or acquisition of data, or analysis and interpretation of data; have been involved in drafting the manuscript or revising it critically for important intellectual content; and have given final approval of the version to be published. PP contributed to the protocol design, study implementation, data analysis, interpretation, writing, and critical review of the manuscript. TLW contributed to protocol design, data analysis, interpretation, writing, and critical review of the manuscript. RK contributed to data analysis, interpretation, and critical review of the manuscript. MJZ contributed to protocol design, study implementation, and critical review of the manuscript. DHK contributed to protocol design, study implementation, and critical review of the manuscript. TMF contributed to protocol design, study implementation, and critical review of the manuscript. GES contributed to protocol design, study implementation, and critical review of the manuscript. KDF contributed to study implementation, interpretation of data, and critical review of the manuscript. EGS contributed to study implementation, interpretation of data, and critical review of the manuscript. VW contributed to study implementation, interpretation of data, and critical review of the manuscript. JAR contributed to the protocol design, study implementation, data analysis, interpretation, writing, and critical review of the manuscript.

## Supplementary Material

Additional file 1 Methodological supplementary material. Click here for file

Additional file 2 Results supplementary material. Click here for file

Additional file 3 **Peyrani Figure Alt 2.** Kaplan-Meier survival curves for safety outcomes. Kaplan-Meier survival curves for each safety outcome using the matched dataset included in Additional file 1. Click here for file

Additional file 4 **Peyrani Figure Alt 3.** Kaplan-Meier survival curves for resource utilization outcomes. Kaplan-Meier survival curves for each resource utilization outcome using the matched dataset included in Additional file 1. Click here for file
