# Supplementary material for: Higher clinical success in patients with ventilator-associated pneumonia due to methicillin-resistant Staphylococcus aureus treated with linezolid compared with vancomycin: results from the IMPACT-HAP study
Source: Crit Care. 2014 Jun 10;18(3):R118. doi: 10.1186/cc13914 (PMC4095575; doi:10.1186/cc13914)
Supplement: Additional file 1 — Methodological supplementary material. [file cc13914-S1.doc]

# Higher Clinical Success in Patients With Ventilator-Associated Pneumonia Due to Methicillin-Resistant *Staphylococcus aureus* Treated With Linezolid Compared With Vancomycin: Results From the IMPACT-HAP Study

**Paula Peyrani, ^1^ Timothy L. Wiemken,^1^ Robert Kelley,^1^ Marcus J. Zervos,^2^ Daniel H. Kett,^3^ Thomas M. File Jr.,^4^ Gary E. Stein,^5^ Kimbal D. Ford,^6^ Ernesto G. Scerpella,^6^ Verna Welch,^6^ Julio A. Ramirez,^1^ and the IMPACT-HAP Study Group**

^1^Division of Infectious Diseases, University of Louisville, Louisville, Kentucky; ^2^Henry Ford Health System, Detroit, MI; ^3^University of Miami Miller School of Medicine and Jackson Memorial Hospital, Miami, Florida; ^4^Summa Health System, Akron, Ohio; ^5^Michigan State University, East Lansing, Michigan; and ^6^Infectious Diseases, Specialty Care Medicines Development Group, Pfizer Inc, Collegeville, Pennsylvania

**Methodological Supplementary Material**

**METHODS**

**Statistical Analysis for Primary Study Outcome***,* **Clinical Success**

All multivariable models were constructed with an initial assessment of variable multicollinearity. As the presence of multicollinearity can be a methodological issue in observational studies, assessing and correcting for its presence is important. The following candidate set of confounding variables was used for all analyses: sex; hospitalization for ≥5 days before ventilator-associated pneumonia (VAP) diagnosis; bronchiectasis; colonization with a multidrug resistant organism; hospitalization for ≥2 days in the prior 90 days; nursing home residence; home infusion therapy; home wound care; active malignancy; end-stage liver disease; chronic obstructive pulmonary disease (COPD); steroid use; presence of any risk factor for a multidrug resistant organism; cardiac disease; renal disease; vascular disease; end-stage renal disease; diabetes; respiratory disease other than COPD; multilobar infiltrates; severe sepsis; appropriate empiric antimicrobial therapy; age; body mass index; Clinical Pulmonary Infection Score (CPIS) at diagnosis; CPIS at day 3 after diagnosis; Acute Physiology and Chronic Health Evaluation (APACHE) II score at diagnosis; hemoglobin at diagnosis; creatinine clearance at diagnosis; and platelet count at diagnosis.

To assess for multicollinearity, correlations between all potential confounding variables were assessed and variables with a correlation of ≥70% were combined. The multicollinearity of variables was further assessed using a linear regression model with success at 14 days as the outcome and all potential confounding variables as predictor variables. Any variable with a variance inflation factor ≥10 was combined with other variables.

Absolute standardized differences were used to assess confounding imbalance between the study arms. Briefly, the absolute standardized difference is the absolute difference between the average of a variable in the treated group and the non-treated group, divided by the standard deviation of the same variable in those groups [1]. The formulas used for the absolute standardized differences were:

*Continuous variables*

| (100(x̅_linezolid_ - x̅_vancomycin)_) ÷ √((σ^2^_linezolid_ + σ^2^_vancomycin_) ÷ 2) |

*Categorical variables*

| (100(*p*_linezolid_ - *p_vancomycin_*)) ÷ √((*p*_linezolid_(1-p_linezolid_) + (*p*_vancomycin_1-p_vancomycin_)) ÷ 2 |

Variables with an absolute standardized difference ≥10% were considered imbalanced. To better balance the dataset, all potential confounding variables mentioned previously were included in a multivariate genetic matching regression model, which matched the data to automatically achieve an optimal covariate balance between the 2 treatment groups [2, 3]. This multivariable matching algorithm used an evolutionary (genetic) search algorithm to weight covariates and match each treated case (linezolid) to the most similar untreated case (vancomycin). Absolute standardized differences for each variable in the matched dataset were then recalculated. Any variable with an absolute standardized difference ≥10 was included in a final logistic regression model using data from the matched dataset. This final model provided odds ratios, *P* values, and 95% confidence intervals (CIs).

Two parallel analyses also were performed. First, we created a model by selecting variables for inclusion in the final regression model using the relative effect statistic (as opposed to the absolute standardized difference). This statistic is a measure of the degree to which a variable confounds the treatment effect on the outcome [4]. The formulas used for the relative effect percent were:

*Continuous variables*

| (βz,x_k_ – βz) ÷ βz) | X 100

*Categorical variables*

| e^(βz,x^_k_ – e^βz)^ ÷ e^βz^) | X 100

where βz,x_k_ and βz are regression coefficients from a generalized linear model fitted as and adjusted model, Y~ Z + X_k_ or an unadjusted model Y~Z. Using this model, variables with a relative effect ≥5 were included in a separate logistic regression model using data from the above matched dataset.

The other analysis was a logistic regression model including all 188 cases. This model adjusted for all potential confounding variables listed previously.

The model with the lowest Akaike information criterion statistic was considered the best-fit model for which results were reported.

*Statistical Analysis for Secondary Study Outcomes*

Kaplan-Meier survival curves were created for the full and matched datasets separately. Results of the complete dataset are depicted in Figures 2 and 3 of the manuscript. Results from the matched dataset are reported in this Appendix (Alternate Figure 2 and Alternate Figure 3).

**References**

1. Austin P. Using the standardized difference to compare the prevalence of a binary variable between two groups in observational research. Communications in Statistics–Simulation and Computation **2009**;38:1228-34.

2. Mebane W, Jr, Sekhon J. Genetic optimization using derivatives: the rgenoud package for R. J Stat Softw **2011**;42:1-26.

3. Ho D, Imai K, King G, Stuart E. MatchIt: nonparametric preprocessing for parametric causal inference. J Stat Softw **2011**;42:1-28.

4. Sampf S. Nonrandom: stratification and matching by the propensity score [R software add-on package]. Version 1.4. Available from: http://CRAN.R-project.org/package=nonrandom. Accessed 11 June 2013.

**Figure Legends**

**Alternate Figure 2.** Kaplan-Meier survival curves in each study arm (matched dataset). *A,* Anemia. *B,* Thrombocytopenia. *C,* Nephrotoxicity.

**Alternate Figure 3.** Kaplan-Meier survival curves in each study arm (matched dataset). *A,* Days on mechanical ventilation. *B,* Length of stay (LOS) in the intensive care unit (ICU). *C,* LOS in the hospital.
