# Supplementary material for: Higher clinical success in patients with ventilator-associated pneumonia due to methicillin-resistant Staphylococcus aureus treated with linezolid compared with vancomycin: results from the IMPACT-HAP study
Source: Crit Care. 2014 Jun 10;18(3):R118. doi: 10.1186/cc13914 (PMC4095575; doi:10.1186/cc13914)
Supplement: Additional file 2 — Results supplementary material. [file cc13914-S2.docx]

# Higher Clinical Success in Patients With Ventilator-Associated Pneumonia Due to Methicillin-Resistant *Staphylococcus aureus* Treated With Linezolid Compared With Vancomycin: Results From the IMPACT-HAP Study

**Paula Peyrani, ^1^ Timothy L. Wiemken,^1^ Robert Kelley,^1^ Marcus J. Zervos,^2^ Daniel H. Kett,^3^ Thomas M. File Jr.,^4^ Gary E. Stein,^5^ Kimbal D. Ford,^6^ Ernesto G. Scerpella,^6^ Verna Welch,^6^ Julio A. Ramirez,^1^ and the IMPACT-HAP Study Group**

^1^Division of Infectious Diseases, University of Louisville, Louisville, Kentucky; ^2^Henry Ford Health System, Detroit, MI; ^3^University of Miami Miller School of Medicine and Jackson Memorial Hospital, Miami, Florida; ^4^Summa Health System, Akron, Ohio; ^5^Michigan State University, East Lansing, Michigan; and ^6^Infectious Diseases, Specialty Care Medicines Development Group, Pfizer Inc, Collegeville, Pennsylvania

**Results Supplementary Material**

**RESULTS**

There was a high correlation (>0.7) between the dialysis and end-stage renal disease variables; thus, these variables were combined for all analyses. No variables had a variance inflation factor ≥10.

A total of 42 patients were not matched using the genetic search algorithm and were dropped from subsequent analyses. After matching using the genetic search algorithm on all variables listed in Table 1 of the manuscript, the following variables remained imbalanced based on the absolute standardized differences and were included in the final regression model: 1) hospitalized for ≥2 days in the prior 90 days; 2) bronchiectasis; 3) hospitalized for ≥5 days before antibiotics for VAP were started; 4) end-stage liver disease; 5) home infusion therapy; 6) COPD; 7) diabetes; 8) risk factors for multidrug-resistant organisms; 9) CPIS at diagnosis; 10) multilobar infiltrates; 11) end-stage lung disease; 12) CPIS 3 days post diagnosis; 13) severe sepsis; 14) APACHE II score; 15) hemoglobin at diagnosis; and 16) creatinine clearance.

Based on the relative effect estimates, the following variable were imbalanced and included in the final regression model: 1) APACHE II score; 2) bronchiectasis; 3) congestive heart failure; 4) body mass index; 5) end-stage liver disease; 6) creatinine clearance at diagnosis; 7) home infusion therapy; 8) severe sepsis; and 9) CPIS at diagnosis.

The Akaike information criterion value for the logistic regression model including the variables imbalanced based on the relative effect estimates (143.1), was smaller than that for the model including the imbalanced variables based on the standardized differences (148.25) and that for the logistic regression model including all 188 cases and all confounding variables (193.64). Therefore, the relative effect adjustment model was defined as the best fit model for which results are reported in the main manuscript. However, the results of the final regression model after matching and the inclusion of variables with an absolute standardized difference ≥10 revealed an odds ratio of 4.40 (95% CI, 1.39–13.90; *P* = 0.001). The relative risk would therefore be 1.32 (95% CI, 1.10–1.42). The adjusted number needed to treat with linezolid to achieve one extra case of clinical success compared with vancomycin was approximately 5. In the logistic regression model including all cases and adjusting for all confounding variables resulted in an odds ratio of 5.07 (95% CI, 1.18–1.41; *P* = .001). The relative risk would therefore be 1.33 (95% CI, 1.18–1.41). The adjusted number needed to treat was approximately 5.

Kaplan-Meier survival curves for each outcome using the matched dataset are included in Supplementary Material (Alternate Figure 2).

Kaplan-Meier survival curves for each outcome using the matched dataset are included in Supplementary Material (Alternate Figure 3).
